# A qualitative interview study on how people with incomplete spinal cord injury experience high-intensity walking exercise

**DOI:** 10.1038/s41394-021-00456-9

**Published:** 2021-10-05

**Authors:** Malene Kolstad Sterling, Matthijs Ferdinand Wouda, Andreas Falck Lahelle

**Affiliations:** 1 Department of Rehabilitation, Asker Municipality, Asker, Norway; 2 Department of Research, Sunnaas Rehabilitation Hospital, Oslo, Norway; 3 Department of Neurology, University Hospital of North Norway, Tromsø, Norway

**Keywords:** Rehabilitation, Rehabilitation

## Abstract

**Study design:**

Qualitative, in-depth research interviews.

**Objective:**

To provide new insight into how people with a recent incomplete spinal cord injury (SCI) experience high-intensity walking exercise after discharge from subacute inpatient rehabilitation.

**Setting:**

Informants for this interview study participated in a previous randomized controlled trial (RCT) that was conducted at Sunnaas Rehabilitation Hospital, Norway.

**Methods:**

Four individual face-to-face interviews were conducted with the participants in natural setting. The interviews were analyzed through systematic text condensation and discussed in the context of experiences of bodily changes.

**Results:**

Four themes emerged that described positive but also challenging bodily experiences related to performing high-intensity walking exercise: “Expectations and motivation”—reasons for participating, “Challenging bodily changes”—impacts on walking ability, “Adaptation strategies”—achieving the high-intensity target level, and “Integrating exercise into a new daily life”—combining participation, *new body* and new life.

**Conclusions:**

This study indicates the importance of participating in a specific exercise program at discharge from subacute inpatient rehabilitation for ambulant people with SCI. However, high-intensity walking exercise may be too demanding to perform during this time period. The insights from the study provide new knowledge that can contribute to improving clinical rehabilitation practice.

## Introduction

### Background

The exercise guideline for adults with spinal cord injury (SCI) promote moderate- to high-intensity aerobic exercise twice a week to improve physical fitness [1]. Exercise programs implemented at early stages of rehabilitation seem to be favorable for the development and maintenance of an active lifestyle [2]. Being physically active is associated with improved life satisfaction [3] and reduces the risk of developing diabetes [4] and cardiovascular diseases [5] among the patient group. The rationale for promoting high-intensity interval training (HIIT) in people with SCI is well established [6]. However, previous studies have focused primarily on upper-body exercises, and there is little evidence for promoting high-intensity exercise involving independent walking [1]. To increase adherence to physical activity, more studies are needed to better understand the effects and experiences of HIIT programs, especially among ambulant people with SCI. It seems likely that a high-intensity program would be experienced differently depending on the exercise mode, injury-specific characteristics such as lesion level and completeness of the injury, as well as the patient’s motivation and life situation.

People with incomplete SCI classified as AIS-D (American Spinal Injury Association Impairment Scale (AIS)) have preserved walking ability [7]. Most of them can perform high-intensity weight-bearing exercises, such as walking uphill [8–10]. It may therefore be favorable to promote weight-bearing exercise to improve physical fitness among this patient subgroup [10]. However, injury to the spinal cord may cause bodily impairments such as muscle weakness and changes in proprioception, which can affect the dynamic walking pattern by impacting coordination, dynamic stride length, balance and gait speed [11–15]. Furthermore, aspects of fatigue, frustration, and loss of control during walking exercises have been reported among this patient group [13, 16]. It seems valuable to incorporate patients’ priorities, preferences and experiences of therapeutic exercise programs throughout the rehabilitation process [17–19]. Thus, there is a need for qualitative studies exploring how ambulant people with SCI experience high-intensity walking exercise.

The present study complements a randomized controlled trial (RCT) [10] by exploring the participants’ experiences of a HIIT program. Such user experiences are recognized as vital components of the rehabilitation process [18] and essential to integrate into research to develop clinical practice [20]. To theoretically interpret perceptions and experiences, we turn to Shaun Gallagher’s framework of the phenomenology of the body [21]. This framework serves as an analytic tool to deepen our understanding of participants’ bodily experiences during physical exercise. Essential components that are relevant to our study are how bodily movements shape the mind through emotions and perceptions, such as motivation, awareness and sense of movement control in people with bodily impairments, and how these perceptions may impact the ability to perform independently walking exercise at high intensity. Gallagher’s phenomenology shares the notion of bodily movements as a continuous interaction between the individual, the task, and the environment [12, 21], which renders the theoretical framework highly relevant for the interpretation of clinical rehabilitation practice.

### Aim and research question

The aim of our study is to provide health professionals with a deeper understanding of patients’ perceptions of high-intensity exercise by addressing the following research question: How do ambulatory people with SCI experience high-intensity aerobic walking immediately after discharge from subacute inpatient rehabilitation? The results may contribute with new knowledge within the field of clinical rehabilitation practice and are potentially transferable to other ambulatory patients with neurological conditions.

## Methods

### Study design

Qualitative in-depth interviews were conducted to explore how ambulant people with SCI experience a high-intensity walking intervention. Qualitative interview designs are grounded in the interpretive research paradigm in which new scientific knowledge is constructed through contextualized human interpretations supported by relevant theory [22]. As flexibility is a cornerstone of qualitative research designs [23], the following sections aim to account for the methodological choices made in this study.

### Context of the study

The informants were recruited from a RCT conducted at Sunnaas Rehabilitation Hospital in Norway that investigated “The effects of moderate- and high-intensity aerobic program in ambulatory subjects with incomplete spinal cord injury” [10]. Ten participants from the RCT conducted a HIIT program consisting of two exercise sessions per week, each lasting for 35 min: 10 min of warming up at 70% of peak heart rate, followed by 4 × 4-min intervals at an intensity of 85–95% of peak heart rate, interspersed with 3 × 3-min recovery periods at an intensity of 70% of peak heart rate. The participants could either walk unaided or run depending on their physical capacity level and impairments. The exercise sessions were carried out in the participants’ home setting, either on a treadmill or outdoors [10].

### Sampling and participants

During the period from spring to fall 2017, five out of the ten participants from the HIIT program were strategically nested by the second author (MFW). All informants had a tetraplegic injury (C2-C6), classified as AIS-D, but they had different injury mechanisms. The characteristics of the informants varied regarding age (30–65), gender (three males and two females), residence, time of injury and compliance with the HIIT program. All five informants signed a written consent form. The first author (MKS) called each participant by phone to set an appointment for the interview. One informant chose to withdraw from the study before conducting the interview.

### Data collection

MKS conducted four individual face-to-face interviews. They were audio-recorded and had a mean duration of 56 min, ranging from 49 to 69 min. The informants chose both the time and place for the interviews. The interviews followed a theme-based and pilot-tested guide with open-ended questions concerning experiences with the intervention, expectations, and significance of participating. Minimal changes were made to the interview guide after the pilot testing. Field notes were made during and after the interviews. MKS imported, transcribed and organized the interviews and field notes in Nvivo11 software, QSR International, London (UK) [24].

### Data analysis

To extract major themes from the data material, we analyzed the interviews by using Malterud’s systematic text condensation [25]. We followed the four steps of the analytic process. Step 1: MKS read through the transcribed interviews multiple times to obtain an initial overview of the material. Data concerning the research question were presented to the third author (AFL), which led to a discussion of possible preliminary themes. Step 2: MKS identified meaning units and fragments of texts from the interviews, discussed them with AFL and coded these units in relation to the theoretical framework and prevailing principles of clinical rehabilitation practice. Step 3: Codes concerning similar contents were sorted into new themes and subgroups. Condensates, short artificial summaries, were written for each subgroup. Step 4: These condensates were discussed among the authors and rewritten into an analytic text, which was validated by carefully comparing it to the transcripts and the original context. Together with illustrative quotes from the interviews, the analytic texts are presented in the results section of this paper. Through the analytic process, themes and subgroups changed as the text developed. The final themes describe both positive and challenging bodily experiences related to performing high-intensity walking exercise after discharge from subacute inpatient rehabilitation.

### Research team and reflexivity

MKS is a neurological physiotherapist with clinical expertise in patients with SCI. AFL is a neurological physiotherapist and senior researcher in qualitative research with experience in phenomenological theoretical frameworks. MFW, both a physiotherapist and movement scientist, is a senior researcher in the field of SCI and exercise physiology. MFW is the first author of the presented RCT [10]. To improve the quality and trustworthiness of the study, the data were analyzed theoretically, critically, and systematically to obtain a balanced presentation of the findings that emphasized both positive and negative experiences.

## Results

Our analysis resulted in four major themes. The first theme describes the informants’ motivation for participating in the HIIT program. The second concerns experiences of bodily changes after the injury that impacted their walking ability. Theme three describes individual strategies during the exercise sessions to achieve the level of high intensity, while the last theme deals with ambitions to integrate exercise into a new daily life.

### Theme one: “Expectations and motivation”

The informants described several positive aspects of participating in the HIIT program. Descriptions of hope, a sense of security and a feeling of being part of a greater context provided them with motivation and courage. A common statement from the informants was that they expected to experience bodily improvements during the intervention period.

“*After the injury, the overall goal was to get as healthy as possible. I hoped participating in the HIIT program would be good for me physically, good for my neck and good in relation to the surgery I had been through. I needed to regain control over my body again*.…”

Several individuals with the same injury classification were included in the HIIT program, which generated a sense of being part of a greater context. They stated that they experienced a sense of security and an assurance that someone was supervising them, which inspired them to maintain the continuity of the exercise sessions. Several informants felt that their functioning improved throughout the intervention period and described experiences of “getting back into shape” and “being exhausted” in a positive sense. They stated that it was motivational to see improvements between their pre- and posttests, which in turn supported their subjective perception of increased physical fitness. Additionally, pushing their bodies during the exercise sessions gave them a positive experience and sense of mastery.

“*It was fun to participate and motivating to see the big improvements during posttests. I think it was great to use the heart rate monitor and physical measurements during the intervention period because I was then able to see so clearly that my fitness level had improved*.…”

### Theme two: “Challenging bodily changes”

The informants stated that their bodily impairments sometimes caused challenges when they tried to achieve 85–95% of their maximal heart rate during the exercise sessions. When they reflected upon these challenges, they often compared their bodily functioning from before the injury with their *new body* after the injury. Several bodily changes were described, such as “muscle weakness”, “reduced balance” and “changes of proprioception” in their truncus and limbs. The informants stated that these bodily impairments impacted their ability to carry out the exercise program and increased their fear of falling during the sessions.

“*It felt like I was lifting my legs higher than what I actually did, so the risk of stumbling or falling was quite high. At discharge from the rehabilitation hospital, the muscles in my legs, the coordination of my body and my balance were not optimal. It felt like my muscles were always tired, and I could not rely on them when I walked*….”

The bodily changes also impacted their overall effort and energy consumption during the exercise sessions. The informants described walking at high intensity as requiring much concentration, which led to increased awareness of their bodily impairments. Movements and activities did not feel as automatic as they did before the injury, and the informants described how they needed to cognitively control their walking pattern during the exercise sessions.

“*I needed to concentrate on being at the correct intensity level, keeping my speed up, and keeping my balance without supporting myself. This took a lot of energy, more energy than before. Before the injury, I could do things without thinking; it was all automatic. However, now, after the injury, all these small tasks require my attention, something I need to bring into focus and use my energy on. I got so tired in my head during the exercise sessions*.…”

### Theme three: “Adaptation strategies”

The informants experienced walking unaided on the treadmill at a high pace as challenging. Their bodily impairments made it necessary to adapt the exercise by use of compensatory strategies to achieve the required level of intensity. They stated that a high pace on the treadmill belt influenced the rhythm and flow of their walking pattern. Some informants needed to compensate for the loss of proprioception by using other somatosensory inputs, such as vision, to achieve control over their bodily movements.

“*It was very demanding to walk on the treadmill. It both challenged my balance and inhibited my movements. I had to follow the speed of the belt instead of following my own preferred walking pace. When I am walking outside, the ground is standing still. On the treadmill, it felt like the whole ground was moving, which forced me to keep my eyes on my feet, which was necessary or else I would fall. I needed to control where I placed my feet on the belt*.…”

Another participant deliberately chose to change the preselected environmental setting by performing the exercise sessions on a stationary bicycle. By doing so, she experienced a better sense of control of her bodily movements and managed to reach the correct heart rate level.

“*When I was bicycling, less attention was needed to balance aspects compared to when I walked on the treadmill. On the bicycle, I could keep focusing on increasing my heart rate. I then had enough energy to follow my pulse, focus on my breathing and the countdown of time*.”

### Theme four: “Integrating exercise into a new daily life”

Some informants described their life after discharge as stressful. They described tension between exercise ambitions, their overall desire for recovery and establishing a new daily life after the injury. Some informants wished that their bodily impairments, such as strength and balance, had been addressed prior to the high-intensity exercise program.

“*When I returned home, it was a lot to deal with. You know, all the daily activities like shopping, cleaning, cooking and so on. I used so much energy on these normal daily activities. In addition, I was supposed to perform this HIIT program, which I had never done before either. For me, it was not the right time to implement this exercise program. I don’t believe the endurance was my main problem after the injury, but I certainly had balance and coordination challenges*….”

However, other informants prioritized the HIIT program because they found that participating in it had positive impacts on being more active in other arenas of their daily life. They described the intervention as tangible and manageable, while other aspects of their new life felt chaotic. They found that the exercise program had become a new daily routine that they might be able to maintain after the intervention period was over.

“*It was great to have a plan, a goal, something specific to focus on.… I will continue to perform the program, maybe not to the same extent, but it has become a new exercise routine in my life*.”

## Discussion

The main findings of this study reveal the informants’ positive and motivational experiences of participating in the HIIT program. However, the informants’ bodily impairments related to their SCI seemed to challenge their balance and walking ability. This negatively influenced the way they experienced exercising at high intensity, especially while walking on the treadmill. Furthermore, the findings reveal a discrepancy between the informants’ preferences regarding rehabilitation after discharge from subacute inpatient rehabilitation and the content and focus of the HIIT program. These findings elucidate the important interactions between the individual, the task and the environment when tailoring specific therapeutic exercise programs for ambulant people with SCI.

Walking was the main task in the HIIT program, which was perceived as a meaningful activity, because it had a social significance and transferability to their everyday life. Experiencing an exercise as meaningful and relevant seems to be important to increase motivation, which is an essential factor in inducing neural plasticity and relearning after an injury [26]. Another motivational factor is experiences of functional improvements during rehabilitation, which may enhance courage to strive for further success and increase ambitions for recovery [13, 15, 26–28]. Our empirical findings indicate that several informants experienced bodily improvements during the intervention period and that the physical exercise tests performed at the hospital were valuable in strengthening these perceptions. The informants’ reflections on “getting back into shape” and “sense of mastery” exemplified that their awareness of perceptions of their bodies was enhanced through the movements. These positive experiences underpin the significance of the connection between bodily movements and thoughts in the performance of activities, which is consistent with other studies [29–31]. From a phenomenology of the body perspective [21], the informants’ perceptions of bodily improvements during the exercise sessions were essential to shaping their cognitive processes, such as motivation, and thereby maintaining the continuity of the exercise sessions. It seems reasonable to propose that considerations of such body-mind connections can be integrated as a useful clinical tool to increase patients’ motivation during rehabilitation and can be transferable to other settings in which functional improvements are obtainable.

Even though participating in the HIIT program enhanced their motivation and bodily improvements, the informants’ experiences of a new body with physical changes impacted their sense of movement control and thereby their capability to perform the exercise sessions on the treadmill. This might indicate that it is too challenging for some ambulant people with SCI to perform unaided walking at a high intensity immediately after discharge from subacute inpatient rehabilitation.

One particular challenge was the perceptions of an impaired body with changes in both sensory and motor systems. The informants repeatedly described “muscle weakness” and “changes in proprioception”, which impacted their balance while walking at high intensity. These individual perceptions are in line with other studies, where bodily impairments seem to reduce the quality of gait by causing loss of movement control during walking exercises [13, 14, 28, 32]. Unaided walking demands adequate strength, endurance, flexibility, balance, and a fine-tuned interaction between the truncus and upper and lower limbs to promote rhythm, flow, and coordination of the walking pattern [12, 33]. As such, the complexity of walking may cause challenges for this patient subgroup due to their injury-specific bodily impairments. Our empirical findings indicate that perceptions of bodily impairments contributed to an increased awareness towards their bodies and movements during the exercise sessions, which generated a sense of a less “automatic” and more “cognitively controlled” walking pattern. Through the perspective of phenomenology of the body, our attention is normally directed away from the body towards the task we are performing [21]. The complexity of walking is therefore processed “in the background” of our awareness, and our attention can be directed towards intentional tasks, such as increasing heart rate to a targeted level. Findings from our study indicate that this was impossible for some informants, as they described the need to divide their attention between both the internal focus on correcting bodily movements and the external focus on the task of walking with high intensity. Such attentional requirements are demanding and seem to exceed the informants’ capability in the early postinjury period. Consequently, attention to correcting bodily movements seems to be the primary priority for some informants, while the main objective of increasing cardiorespiratory fitness by performing the HIIT program becomes secondary.

Another challenge that affected the informants’ experience of performing the HIIT program was the preselected environmental setting. Some informants stated a weakened feeling of “being in charge” of their movements and explained that the treadmill posed inconveniences and constraints. From a phenomenology of the body perspective, a reduced sense of agency may occur when individuals experience diminished control over initiating bodily movements [21]. Several informants in our study described that they had to use compensatory strategies to respond to the moving belt on the treadmill. The informants’ problem-solving technique of adapting their walking pattern or relying on visual input might be beneficial for their walking performance. However, becoming dependent on such compensatory strategies during the early stages of recovery may delay or hinder the development of selective motor control, which is important for regaining optimal selective movements and thereby activity [33]. As such, overground walking might be a better alternative for some ambulant people with SCI.

In contrast to our findings, several other studies emphasize perceptions of improvements in walking pattern, balance, and better sense of control over bodily movements by participating in walking exercises [34–38]. However, in these studies the walking exercises were supervised and participants walked with either harness or exoskeletons. This is an essential difference compared to our study in which participants exercised without any aids or support and without supervision.

Considering these previously discussed elements, unaided walking at high intensity on a treadmill at early stages of rehabilitation is not optimal for all ambulant people with SCI. We suggest that high-intensity exercise programs should be individually adjusted, with consideration of subjective bodily experiences, and the approach should be gradually adapted to individual needs. In clinical rehabilitation practice, it seems to be advantageous to combine strength, balance, task-specific walking, and aerobic exercises to increase physical fitness and neural plasticity [39]. Health professionals need to recognize the fundamental perceptual and physical individual challenges of bodily change after an injury if they are to implement effective exercise programs among ambulant people with SCI.

### Limitations

The small number of informants is a considerable limitation of the study because more informants might have provided additional elements [23, 40]. Due to limited resources in this study, only 5 out of 10 participants from the RCT were recruited in the interview study. It is likely that other persons with incomplete SCI might have different experiences with high-intensity exercise than those described in this current study. Regardless of the small number of informants, they have elucidated in-depth aspects of both positive and challenging experiences. Such rich descriptions are considered as trustworthy data in qualitative research and acknowledged as more important than sample size [23, 41]. The fact that all informants had a cervical injury decreases the external validity of the results. Paraplegics might experience walking ability differently due to their increased control of the trunk and upper limbs. However, ambulatory people with a cervical SCI comprise a substantial part of the Norwegian SCI population [42].

## Conclusion

This study emphasizes how high-intensity walking exercise is experienced among ambulant people with SCI immediately after discharge from subacute inpatient rehabilitation. Implementation of a specific exercise program, including polyclinical follow-up testing and experiencing physical improvements, seems to enhance motivation and participation. However, our findings indicate that individual postinjury bodily impairments negatively influence the informants’ feeling of movement control, which seems to impact their ability to walk at high intensity on a treadmill. If future studies are to implement effective exercise programs with the goal of increasing cardiorespiratory fitness, the participants’ experiences of bodily changes during the exercise session should be taken into account. To improve clinical rehabilitation practice, we suggest that aerobic exercises should be tailored to each patient’s impairment level and individual needs.
